# Dr. Isaac E. Bennett

**Published:** 1914-04

**Authors:** 


Dr. Isaac E. Bennett, Buffalo, 1872, died at his home in Plano, Ill., February 19, aged 66.

